# The Impact of Robot-Assisted Digital Education on Prenatal Women's Health Literacy: A Randomized Controlled Trial

**DOI:** 10.1155/jonm/8865606

**Published:** 2025-06-05

**Authors:** Chin-Lan Yang, Zi-Yin Xu, Ching-Yi Chang

**Affiliations:** ^1^Department of the Nursing, Hsin Sheng Junior College of Medical Care and Management, Longtan District, Taoyuan City, Taiwan; ^2^School of Nursing, College of Nursing, Taipei Medical University, 250 Wuxing Street, Taipei City 11031, Taiwan

**Keywords:** gestational diabetes mellitus, health literacy, robot-assisted digital education, robot-assisted learning

## Abstract

**Objective:** This study aimed to evaluate the effectiveness of a robot-assisted digital education method in reducing anxiety and promoting healthy behaviors among pregnant women with gestational diabetes mellitus (GDM).

**Design:** A randomized controlled trial was conducted to compare the impact of robot-assisted health education with conventional video-based education.

**Participants:** A total of 66 pregnant women from a single hospital were randomly assigned to either the experimental group (*n* = 32) or the control group (*n* = 34).

**Outcomes:** The primary outcome was the reduction of anxiety levels. Secondary outcomes included health education satisfaction, health literacy, and acceptance of technology.

**Results:** The results indicated that the robot-assisted digital education method significantly reduced anxiety levels among pregnant women. Additionally, participants in the experimental group reported higher satisfaction with health education, improved health literacy, and greater acceptance of technology compared to the control group.

**Conclusion:** This study highlights the potential benefits of integrating robotic technology into health education for pregnant women. In the global information age, the findings provide valuable insights for educators and researchers in medical institutions aiming to enhance long-term health education through innovative digital tools.

**Trial Registration:** ClinicalTrials.gov identifier: NCT06999421

## 1. Introduction

In Taiwan, under the National Health Insurance (NHI) benefits, every pregnant woman is entitled to 14 free prenatal examinations. These examinations include essential procedures such as gestational diabetes mellitus (GDM) screening, which allows for the early detection of fetal abnormalities. Historically, this health information has been provided to pregnant women by nurses, requiring at least a 20 min explanation for each patient to understand fully. However, owing to the shortage of nursing labor, there is a new issue of whether standardized GDM health information can be accurately conveyed to pregnant women through digital systems or tools [1]. Beyond disseminating GDM health information, it is expected that digital systems could serve as a reference for educational training in process reform as well as an assistant to reduce the load of nursing staff.

Researchers have recognized the potential of using digital systems or tools in health education in terms of enhancing learning outcomes, enjoyment of the learning process, and learners' capacity to integrate health information [2, 3]. Advances in emergent technologies, such as robots, have further spurred innovative changes in teaching and learning across various fields, allowing researchers to reconsider more efficient and effective ways for delivering health knowledge [4]. In recent years, robot-assisted education has shown significant benefits compared to traditional methods. Studies have highlighted that the use of robots in education can enhance student learning by improving engagement, facilitating remote learning, and enhancing knowledge [5]. Additionally, research on culture education using robots has demonstrated that users, both adult and nonadult, have expressed higher satisfaction levels with the educational effectiveness and significant value of robot-mediated systems, emphasizing the importance of educational function and system value [6].

Thus, the design of intelligent education robots has also been highlighted for its convenience, flexibility, and ability to improve teaching effectiveness through features such as display screens, voice players, cameras, and projectors [7]. Robot-assisted digital education has shown significant potential in improving health literacy, as evidenced by studies focusing on different demographics. Research by Wei et al. [8] demonstrated that robot-assisted learning significantly enhances health knowledge, literacy, motivation, and learning perception. Furthermore, scholars have emphasized the importance of interactive computer programs for empowering communication among women with limited literacy skills, showcasing improved engagement and information disclosure during prenatal visits [9]. These findings collectively suggest that robot-assisted digital education can play a crucial role in enhancing health literacy among prenatal women, offering innovative and effective approaches to education and empowerment.

This study introduced a robot-assisted education program, specifically designed to digitally deliver content and enhance interactive communication in nursing care, with the objective of potentially alleviating the anxiety linked to the ambiguity in the blood glucose testing process for GDM. The developed educational robots aimed to disseminate information about GDM and evaluate outpatient pregnant women's satisfaction with the provided guidance, as well as their health literacy, anxiety levels, and technology acceptance. This exploration not only highlights the potential of educational robots to enhance the effectiveness of health education but also provides patients with new perspectives and methods for reducing anxiety. To evaluate the effectiveness of the implemented approach, the following research question was proposed:

1. Does robot-assisted digital education improve prenatal women's satisfaction, health literacy, anxiety levels, and technology acceptance compared to the conventional approach?
2. What other factors of robot-assisted digital education influence the health literacy of prenatal women?

## 2. Literature Review

Health knowledge is typically disseminated through various technologies, including smartphones, tablets, and the Internet of Things. Enhancing individuals' health literacy is a key step toward achieving a healthier society. Scholars have indicated that improved health literacy not only benefits individual well-being but also contributes to improving public health standards while helping to reduce overall healthcare costs [10, 11]. High levels of health literacy are crucial for comprehending and following public health recommendations that aid in disease prevention and enhance the efficiency of medical services [12, 13]. Appropriate health literacy also allows individuals to more effectively assess the reliability of sources of information, reducing the impact of misinformation [14]. Therefore, enhancing the health literacy of pregnant women empowers them with greater autonomy by enabling them to participate in decision-making processes related to their healthcare, including choosing treatment options and managing their pregnancy [15].

According to previous research by Li et al. [16], the risk of developing GDM is positively correlated with increasing maternal age. The prevalence of oral glucose tolerance test (OGTT) positivity and the GDM value were 20% and 2.2%, respectively, for women aged 25 years or younger. The values increased to 37.8% and 14.7% for women over the age of 35 years. Multiparous women also have a higher risk of developing GDM, with the incidence rates of OGTT positivity and the GDM value increasing with the number of pregnancies [17]. For women in their first pregnancy, the rates of OGTT positivity and the GDM value were 21.2% and 3.5%, respectively; for women with more than four pregnancies, the rates increased to 37.5% and 14.6%. Glucose from the mother can pass through the placenta into the fetus, but insulin cannot. Upon entering the second trimester, hyperglycemia stimulates the fetus to secrete insulin. Poblete and Olmos reported that the hyperglycemia and hyperinsulinemia associated with GDM could stimulate the fetal pancreas to secrete excessive insulin, leading to excessive nutrients entering the fetus and fetal overgrowth and adiposity, with 15%–45% of GDM cases complicated by macrosomia [18]. An accumulation of fat in the abdomen and shoulders of the fetus increases the risk of shoulder dystocia and fetal injury.

Through the innovative integration of new technology, the application of educational robots is expected to assist nursing staff in conveying health knowledge consistently and effectively within the limited timeframes of clinical environments, which would also help to standardize health education [19]. There have been several studies on health knowledge dissemination through robot-assisted teaching and medical care, where robots have been shown to provide sufficient health information on medical cases [20, 21]. As a technology capable of interacting fluidly with humans, robots have integrated functions that enable clear knowledge transmission. Friendly user interfaces and interactive functions can enhance patients' acceptance of technology during health education, thereby increasing their willingness and interest in using technology for health management [22].

By embedding educational materials into robots, robot-assisted learning makes use of the pleasant nature of interactions between robots and learners and has been proven to be an effective teaching method [23]. For example, robot-assisted education can reduce patients' anxiety levels. Chang et al. used cute robots to explain upcoming medical procedures to children, creating a relaxed and enjoyable learning environment [24]. This greatly enhanced the learners' participation and emotional investment in learning, which helped to reduce their anxiety and increase their satisfaction with the learning process. These kinds of robot-based interventions in nursing can maintain individuals' attention during interactions, which helps to shift emotions and alleviate anxiety about unknown examinations.

The use of educational robots in outpatient clinics to disseminate pregnancy-related health information has not yet been tested. To address this gap, this study developed and deployed educational robots equipped with health information about GDM, followed by field verification of participating pregnant women's learning performance. This study can serve as an empirical reference for the future development of various educational robots to facilitate health education.

## 3. Methods

### 3.1. Study Design and Setting

This study aimed to present research findings in accordance with the CONSORT guidelines for RCTs and to adhere to the TIDieR checklist. This study used convenience sampling to include all outpatient cases and routine prenatal checkups for GDM in pregnant women. Eligibility criteria for participants included the absence of chronic medical conditions, age over 21 years, local Taiwanese, and GDM for pregnant women with a normal initial fetal examination during prenatal assessment.

A randomized experimental design was implemented, incorporating a robot-assisted digital health education approach led by qualified medical staff to disseminate GDM-related information to pregnant women. The robot-assisted digital health system, developed by an engineer, featured content tailored to individual needs (e.g., adjusted according to patient-specific requirements). To ensure the system's reliability and validity, it was evaluated by two medical staff members and an obstetrics and gynecology specialist. The kappa value between the two medical staff ratings was 0.90, indicating a high degree of consistency.

The experiment was conducted in the obstetrics outpatient clinic of a regional hospital from November 2023 to January 2024, with no modifications to the robot or intervention methods to ensure consistent inclusion criteria. Consistency in the intervention and data collection was ensured by the same researcher, who had 2 years of clinical experience in obstetrics and had received research training. In addition, both researchers and participants were blinded to group assignments.

For the *t*-test sample size calculation, G∗Power was used with effect sizes of 0.2, 0.5, and 0.8 for small, medium, and large effects, respectively. This study applied a medium effect size (0.5), requiring at least 64 samples. Therefore, a total of 80 women undergoing outpatient prenatal examinations were recruited at the same hospital and volunteered to join the study. Participants volunteered to participate in the study and completed a consent form, which was approved by the institution ethics committee. Participants were informed that they could withdraw their participation at any time without affecting their rights to prenatal care.

Participants were randomly divided into control and intervention groups using a computer-based randomization method that prevented researchers from predicting or influencing group allocation. The control group received tablet-based medical education, while the experimental group received education through robots, with comprehensive support from nurses and doctors. These healthcare professionals assisted patients in interacting with the robots and actively participated in guiding and clarifying the educational content. Moreover, the educational content via GDM for the control group was identical to that of the experimental group to ensure study fairness and to maintain equivalent involvement of healthcare professionals across both groups. After receiving the health education, a survey was conducted to investigate learner satisfaction, health literacy, anxiety levels, and technology acceptance, as well as any correlations with the data collected during recruitment.

### 3.2. Development of Robot-Assisted Digital GDM Education Program

Following the methodology of Chang [24], this study programmed educational GDM information as well as various emotional expressions, gestures, and sounds into a robot. The robot featured a facial screen that served as an interface for playing videos or applications. Its screen could also display various emotional expressions, its speakers played sounds, and the robot itself performed gestures to interact with patients.

In this study, the robot's educational content focused on GDM, teaching pregnant women about the impact of GDM on pregnancy, precautions, and the examination process. The video content was designed as cartoon animations, and questions were posed during specific story segments to ensure correct understanding of the content. Through interactive scenarios and storylines, patients could repeatedly interact with the robot to acquire the correct knowledge or ask for more details during the nursing and treatment process. This friendly interactive design was intended to reduce GDM-associated anxiety.

### 3.3. Experimental Procedure

After explaining the research purpose regarding GDM education, the experimental group participated in a 30 min robot-assisted digital education program, while the control group received the same GDM health education via a 30 min tablet-based video. Both groups of women undergoing prenatal examinations thus received the same GDM information and educational content during the consultation; the primary difference lay in the interactivity of the robot-assisted digital education method, where the robot displayed additional gestures and facial expressions, and the women could interact by clicking on the robot's screen or by using voice feedback. By contrast, the control group interacted with the video education content via a tablet. After the 30 min educational consultation, participants were asked to complete a Google Form questionnaire.

### 3.4. Instruments

Health education satisfaction was evaluated with a questionnaire adapted from a survey by Black et al. using a Likert scale ranging from 1 (*very satisfied*) to 5 (*very dissatisfied*) as well as an open-ended question [25]. Health literacy was assessed using the Health Literacy Questionnaire (HLQ) described by Wahl et al., which also used a Likert scale ranging from 1 (*strongly disagree*) to 5 (*strongly agree*) [26]. Anxiety levels were assessed using the Hamilton Anxiety Scale (HAMA), which was developed by Hamilton, using a Likert scale from 1 (*no symptoms*) to 5 (*severe anxiety*) [27]. Referencing Hwang et al., the technology acceptance of the pregnant women was measured using another Likert scale ranging from 1 (*strongly agree*) to 5 (*strongly disagree*) [28]. Cronbach's alpha values for these scales ranged from 0.80 to 0.84, indicating a good level of internal consistency among the items within each questionnaire.

### 3.5. Ethical Statement

The study was performed in line with the principles of the Declaration of Helsinki. Approval was granted by the institutional review board of Taipei Medical University.

## 4. Results

Data were analyzed using SPSS 25 (IBM, Armonk, NY, USA). To compare the categorical variables of the two groups, an independent samples *t* test and the one-way ANOVA were conducted.

### 4.1. Participant Characteristics

**Figure 1:**
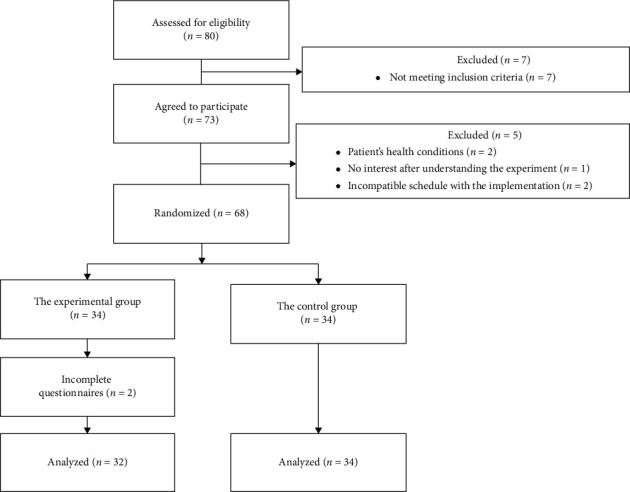
Participant selection flow chart.

Figure 1 shows a flowchart for participant selection. Based on the inclusion criteria, seven women did not meet the participation qualifications as they were not citizens, and five women refused to participate due to their health condition, a lack of interest after understanding the experiment, or scheduling issues. The remaining 68 pregnant women signed the consent form and agreed to participate in the experiment. The participating pregnant women were randomly assigned to either the experimental group (*n* = 34) or the control group (*n* = 34), and their baseline characteristics were surveyed via a questionnaire, including age, gravidity, gestational age, previous delivery experience, prenatal education, and expected method of delivery. Two participants in the experimental group did not complete the postintervention Google Form questionnaire, bringing the number of participants in the experimental group to 32 and the total number of participants to 66, with an actual participation rate of 82.5%. All other participants completed the surveys.

In this study, the average age of the 66 surveyed pregnant women was 34.25 years old (standard deviation: 4.33 years), with a slight majority under 35 years old (*n* = 34, 51.5%), and the remaining participants aged 35 years and older (*n* = 32, 48.5%). The average number of pregnancies of the participants was 1.73 (standard deviation: 0.94); primiparas (first-time pregnant women) were slightly more common (*n* = 34, 51.5%), and multiparas (women who have been pregnant more than once) were slightly less common (*n* = 32, 48.5%). The average gestational week of the pregnant women was 19.79 weeks (standard deviation: 10.08 weeks), with the majority in the second trimester (13–29 weeks; *n* = 28, 42.4%), followed by those in the first trimester (0–12 weeks; *n* = 21, 31.8%) and then those in their third trimester (30 weeks or more; *n* = 17, 25.8%). Regarding their previous childbirth experiences, the majority had primipara experience (*n* = 34, 51.5%), followed by those who had experienced vaginal delivery (*n* = 16, 24.2%) and those who had a cesarean section (*n* = 16, 24.2%). The majority of participants had not participated in any prenatal education courses (*n* = 50, 75.8%); the remainder had taken a prenatal education course (*n* = 16, 24.2%). The expected method of delivery was predominantly natural birth (*n* = 54, 81.8%), with cesarean sections being less common (*n* = 12, 18.2%).

Using the chi-squared test, Fisher's exact test, or independent samples *t* test, a significant difference in the number of pregnancies between the two groups was found (*χ*^2^ = 4.95, *p*=0.026). The control group had a higher proportion of multiparas (61.8%) compared to the experimental group (34.4%). No significant differences were found in terms of age, gestational weeks, previous childbirth experience, prenatal education course history, or expected method of delivery (*p* > 0.05), indicating a homogeneous distribution of most basic demographic characteristics between the two groups (Table 1).

### 4.2. Health Education Satisfaction, Health Literacy, Anxiety, and Technology Acceptance Between Groups

The independent samples *t* test was used to compare the differences between the experimental group and the control group in terms of their health education satisfaction, health literacy, anxiety levels, and technology acceptance to understand if there were significant differences in the effectiveness of the robot-assisted program and the tablet-based education.  Table 2 shows that posttest learning satisfaction reached significance (*t* = −0.74, *p*=0.043). Health literacy, however, did not exhibit a significant difference between the two groups following the intervention (*t* = −0.96, *p*=0.341). Anxiety levels were significantly different between the two groups after the intervention (*t* = −2.06, *p*=0.046). A comparison of the means revealed that the experimental group had lower anxiety levels compared with the control group, providing evidence that the robot-assisted education was more effective than the tablet-based education at reducing anxiety. In terms of technology acceptance, no significant difference was found between the two groups of pregnant women following the intervention (*t* = −0.31, *p*=0.760).

### 4.3. Health Literacy

Table 3 shows that pregnant women in the experimental group exhibited significant associations between health literacy and the baseline characteristics of current gestational weeks and prenatal education course history (*p* < 0.05). A further comparison among the experimental group revealed that the health literacy of women in their second trimester (13–29 weeks) was superior to that of women in their third trimester (30 weeks or more); the health literacy of those who had taken prenatal education courses was also superior to those who had not participated in such courses. No significant association was found between health literacy and age, number of pregnancies, previous childbirth experience, or expected method of delivery.

In the control group, no significant association was found between health literacy and the baseline characteristics of age, number of pregnancies, current gestational weeks, previous childbirth experience, prenatal education course history, or expected method of delivery (*p* > 0.05).

## 5. Discussion

The present study implemented a robot-assisted digital education program to teach prenatal women. A randomized controlled experiment was conducted to examine the effectiveness of the approach. A significant difference was found in health education satisfaction between the experimental and control groups after the intervention. In the experimental group, baseline characteristics, including age, number of pregnancies, gestational weeks, previous childbirth experience, prenatal education course history, and expected method of delivery, exhibited a significant association with their satisfaction with the health guidance provided (*p* < 0.05). Further comparisons revealed that satisfaction levels were higher for pregnant women in the early (0–12 weeks) and mid-pregnancy (13–29 weeks) stages of pregnancy compared to those in late pregnancy (30 weeks or more). In the control group, those who had not taken previous prenatal education courses had higher levels of satisfaction than those who had already participated in such courses, while other baseline characteristics including age, number of pregnancies, previous childbirth experience, expected method of delivery, and current gestational weeks did not show a significant association (*p* > 0.05).

The observed results were consistent with those of Chung, who found that implementation of an appropriate education strategy by health staff during prenatal consultation showed great potential for assisting pregnant women with a smoother birth process [29]. Chang et al. also showed that robot-assisted digital education could significantly affect patients' emotions during nursing care [24].

In terms of health literacy among the pregnant women in the experimental group, the baseline characteristics of age, number of pregnancies, current gestational weeks, previous childbirth experience, prenatal education course history, and expected method of delivery showed no significant association with health literacy (*p* > 0.05). In the control group, the baseline characteristics of gestational weeks and prenatal education course history showed a significant association with health literacy (*p* < 0.05). Further comparisons in the control group revealed that the health literacy of women in their second trimester (13–29 weeks) was superior to that of women in their third trimester (30 weeks or more), and those who had not taken a prenatal education course had higher health literacy levels compared with those who had participated in such courses. Age, number of pregnancies, previous childbirth experience, and expected method of delivery showed no significant association with health literacy. These findings suggest that any educational approach could improve clients' health literacy, whether through robot-assisted or video education, which both allowed them to read materials and interact to gain knowledge related to GDM. The observed results aligned with the findings of Zibellini et al. and RobatSarpooshi et al., who reported that individuals engaging in self-care behaviors, such as participating in educational courses early in pregnancy, exhibit higher levels of health literacy [30, 31].

Anxiety levels among pregnant women in the experimental group were found to be significantly associated with having taken a prenatal education course. A further comparison revealed that the anxiety levels of those in the experimental group who had not participated in prenatal education courses were higher than those who had. Age, number of pregnancies, current gestational weeks, previous childbirth experience, and expected method of delivery showed no significant association with anxiety levels (*p* > 0.05). In the control group, no significant association was found between anxiety levels and any baseline characteristics (*p* > 0.05). These findings aligned with those of Connolly et al. [19], who reported that robots are beneficial for learner participation. In terms of daily health education in medical environments, using a digital technology-based educational approach, such as the robot-assisted approach, could help patients learn the information delivered by medical personnel and allow them to engage in more interactive health education activities [32]. Rasouli et al. also revealed that robots could stimulate a reduction in anxiety and help patients participate more fully in learning activities [33]. The findings of this study add support to this argument, since the integration of a robot enabled patients to learn and be more involved in the educational activities.

In the experimental group, technology acceptance was shown to have no significant association with any of the baseline characteristics (*p* > 0.05). For the control group, current gestational weeks and prenatal education course history were found to be significantly associated with technology acceptance (*p* < 0.05). Further comparisons revealed that technology acceptance was higher for women in their second trimester (13–29 weeks) compared with those in late pregnancy (30 weeks or more), and those who had not participated in a prenatal education course had higher levels of technology acceptance than those who had participated. Age, number of pregnancies, previous childbirth experience, and expected method of delivery showed no significant association with levels of technology acceptance. These results suggest the need for longer study periods and larger sample sizes to verify the impact of the robot-assisted digital education approach in different contexts.

The implemented method promotes health education interactivity via robot-assisted digital education. Previous research has highlighted the importance of fostering interactive communication [34]; in most clinical settings, pregnant women have almost no opportunity to participate in interactive health education. For instance, in video-based health education, healthcare professionals play videos without allowing pregnant women to operate or interact with them directly, which does not encourage learning engagement. By contrast, the method proposed in this study allowed pregnant women to interact with robots through a touch screen, offering a stark contrast to video health education where the direct observation of videos is the only form of learning. Additionally, this study found that fears and anxieties about the hospital environment were reduced in pregnant women after learning health information through interesting content and playing with the robot. Moreover, healthcare professionals and accompanying persons can design interactive personalized questions based on different health education content and embed them in the robot. This approach can reduce anxiety during prenatal waiting periods and improve the quality of medical visits.

The present findings confirmed that the robot-assisted digital education method effectively improved the educational satisfaction of pregnant women while also reducing their anxiety about GDM. To address the challenges related to globalization and health education, these positive findings provide a useful reference for nursing educators attempting to apply digital technologies. Patrício et al. suggested that nursing educators must develop innovative health education strategies centered on the patient to enhance patients' and their families' health literacy, which can subsequently improve their health behaviors and healthcare capabilities [35]. The method proposed in this study offers an excellent clinical application of a new health strategy and highlights its potential for broad adoption by nursing educators in other fields. This approach can also be applied to health education programs in different scenarios and for various issues, such as explaining home rehabilitation processes, necessary examinations, and important physiological monitoring during invasive inspections. It can also be used for patients undergoing surgical treatment, teaching them and their families coping skills or how to collaborate with their peers to solve daily healthcare problems.

### 5.1. Implications for Nursing Management

Integrating robot-assisted digital education into patient education programs can significantly reduce anxiety levels among pregnant women. The study highlights that digital education methods enhance health education satisfaction. By incorporating robot-assisted education, pregnant women showed improved health literacy. Moreover, the acceptance of technology by pregnant women as indicated by the study suggests that nursing managers need to invest in training and resources to ensure both staff and patients are comfortable and proficient with new technological tools. In the global information age, the ability to track long-term health education through technological tools becomes crucial.

To support the integration of robot-assisted digital education, nursing managers must allocate appropriate resources, including funding for technology, training for staff, and continuous evaluation of the education programs. Nursing management should collaborate with educators and researchers at medical institutions to leverage insights and advancements in technological health education. This collaboration can help in refining and expanding the use of digital tools in patient education. By addressing these implications, nursing management can enhance the quality of care, improve patient outcomes, and stay at the forefront of healthcare education innovation. This implies not just a one-time intervention but also a sustained effort to incorporate technology-based educational tools into routine care.

## 6. Conclusions

This study adopted a robot-assisted digital education approach to support the health education of pregnant women and explored its impact on their learning satisfaction, health literacy, anxiety levels, and technology acceptance. The findings showed that this method can effectively enhance pregnant women's health literacy, which was especially evident among those who had not already participated in a prenatal education course. This indicated that robot-assisted digital and video education, along with allowing the pregnant women to read materials and interact with a learning platform, could increase their knowledge related to GDM. In terms of anxiety levels, only the women in the experimental group who had already participated in a prenatal education course showed a significant association, with a further comparison showing lower levels of anxiety among those who had not previously participated in a course. These findings support the view that using robot-assisted digital technology education methods can help pregnant women learn the information provided by healthcare professionals, which enables them to participate more in health education activities.

While robot-assisted digital education showed positive effects on pregnant women's health literacy, anxiety, and technology acceptance, challenges remain in terms of costs and the additional time needed to prepare the health education materials. Moreover, there are some limitations in the present study: (1) Only 66 pregnant women from a single hospital were included in the study, which might not be able to fully reflect the wider population, implying the need to perceive the findings in a conservative manner. (2) The study period was not long, implying that the effects of long-term use of robot-assisted digital education on pregnant women's health behaviors and anxiety remain an open issue. (3) The study partially met the TIDieR criteria (e.g., tailoring GDM content based on the individual needs of pregnant women at the time of enrollment), implying that better adherence to these criteria could enhance its contribution. (4) The study acknowledged that participants' anxiety may have been influenced by self-awareness or social desirability bias, which was not measured prior to the intervention. Therefore, future studies should measure anxiety at both baseline and postintervention to enable within-group comparisons and attribute changes to the intervention. (5) Two participants in the experimental group were lost to follow-up and were excluded from the analysis. Future intention-to-treat (ITT) analyses should include participants who did not complete the survey, with missing data addressed through imputation methods such as multiple imputation or last observation carried forward. (6) This study explored how different participant characteristics interacted with each educational method (robot-assisted vs. tablet-based). However, the study's approach may limit the generalizability of findings, as group-specific effects may not fully capture trends in the overall population. Future studies should consider conducting both per-group and overall analyses to provide a more comprehensive understanding of these associations.

These limitations suggest the need for long-term follow-up research with a larger sample size to verify the effectiveness of applying this method to different areas of medical care. It is also suggested to further investigate the effectiveness of robot-assisted digital education by considering additional factors to provide new insights, such as analyzing pregnant women and their families' interactive behaviors or interview results to deepen understanding of the therapeutic communication, emotional involvement, and anxiety conditions among pregnant women.

## Figures and Tables

**Table 1:** Participant characteristics (*N* = 66).

Characteristics	(*N* = 66)	Groups		*t*/*χ*^2^	*p*
		Experimental group (*n* = 32)	Control group (*n* = 34)		
Age^a^	34.25 ± 4.33	34.53 ± 4.71	33.99 ± 4.00	*t* = −0.50	0.617
Under 35 years old	34 (51.5)	14 (43.8)	20 (58.8)		
Over 35 years old	32 (48.5)	18 (56.3)	14 (41.2)		
Gravidity^b^	1.73 ± 0.94	1.47 ± 0.76	1.97 ± 1.03	*χ* ^2^ = 4.95^∗^	0.026
Primipara	34 (51.5)	21 (65.6)	13 (38.2)		
Multiparous	32 (48.5)	11 (34.4)	21 (61.8)		
Gestational age^b^	19.79 ± 10.08	19.47 ± 10.66	20.09 ± 9.66	*χ* ^2^ = 1.00	0.607
First trimester (0–12 weeks)	21 (31.8)	12 (37.5)	9 (26.5)		
Second trimester (13–29 weeks)	28 (42.4)	12 (37.5)	16 (47.1)		
Third trimester (30 weeks and beyond)	17 (25.8)	8 (25.0)	9 (26.5)		
Previous delivery experience^c^					0.471
Vaginal delivery	16 (24.2)	6 (18.8)	10 (29.4)		
Caesarean section	16 (24.2)	3 (9.4)	13 (38.2)		
Primipara (primigravida)	34 (51.5)	23 (71.9)	11 (32.4)		
Prenatal education^b^				*χ* ^2^ = 0.19	0.663
Yes	16 (24.2)	7 (21.9)	9 (26.5)		
None	50 (75.8)	25 (78.1)	25 (73.5)		
Expected method of delivery^b^				*χ* ^2^ = 0.27	0.601
Vaginal delivery	54 (81.8)	27 (84.4)	27 (79.4)		
Caesarean section	12 (18.2)	5 (15.6)	7 (20.6)		

*Note:* Descriptive statistics for each group are presented as the mean ± standard deviation.

^a^Independent samples *t* test.

^b^One-way ANOVA.

^c^Fisher's exact test.

^∗^ *p* < 0.05.

^∗∗^ *p* < 0.01.

^∗∗∗^ *p* < 0.001.

**Table 2:** Differences in study variables between the experimental and control groups.

Variables	Groups		*t*	*p*
	Experimental group (*n* = 32)	Control group (*n* = 34)		
Health education satisfaction	4.46 ± 0.62	4.57 ± 0.55	−0.74^∗^	0.043
Health literacy	4.42 ± 0.61	4.56 ± 0.52	−0.96	0.341
Anxiety	1.08 ± 0.18	1.24 ± 0.41	−2.06^∗^	0.046
Technology acceptance	4.35 ± 0.68	4.40 ± 0.53	−0.31	0.760

*Note:* Data are presented as the mean ± standard deviation.

^∗^ *p* < 0.05.

^∗∗^ *p* < 0.01.

^∗∗∗^ *p* < 0.001.

**Table 3:** Associations between baseline characteristics and health literacy.

Characteristics	Experimental group					Control group				
	*N*	M ± SD	*F*/*t*	*p*	Post hoc	*N*	M ± SD	*F*/*t*	*p*	Post hoc
Age^a^			0.15	0.882				0.64	0.526	
Under 35 years old	14	4.57 ± 0.54				20	4.48 ± 0.68			
Over 35 years old	18	4.54 ± 0.53				14	4.34 ± 0.50			
Gravidity^a^			1.19	0.241				0.43	0.673	
Primipara	21	4.63 ± 0.49				13	4.48 ± 0.66			
Multiparous	11	4.40 ± 0.58				21	4.39 ± 0.58			
Gestational age^b^			5.60^∗∗^	0.009	2 > 3			0.40	0.674	
First trimester (0–12 weeks)	12	4.53 ± 0.50				9	4.54 ± 0.48			
Second trimester (13–29 weeks)	12	4.85 ± 0.30				16	4.43 ± 0.69			
Third trimester (30 weeks and beyond)	8	4.15 ± 0.58				9	4.28 ± 0.60			
Previous delivery experience^c^			2.84	0.074				0.10	0.902	
Vaginal delivery	6	4.83 ± 0.36				10	4.49 ± 0.52			
Caesarean section	3	4.00 ± 0.00				5	4.44 ± 0.52			
Primipara (primigravida)	23	4.56 ± 0.54				19	4.38 ± 0.69			
Prenatal education^b^			−2.82^∗∗^	0.009				1.36	0.183	
Yes	7	4.11 ± 0.44				9	4.65 ± 0.42			
None	25	4.68 ± 0.48				25	4.34 ± 0.65			
Expected method of delivery^b^			0.72	0.478				1.55	0.130	
Vaginal delivery	27	4.58 ± 0.52				27	4.50 ± 0.54			
Caesarean section	5	4.40 ± 0.55				7	4.11 ± 0.77			

*Note:* Descriptive statistics for each group are presented as the mean ± standard deviation.

^a^Independent sample *t* test.

^b^One-way ANOVA.

^∗^ *p* < 0.05.

^∗∗^ *p* < 0.01.

^∗∗∗^ *p* < 0.001.

## Data Availability

The data that support the findings of this study are available from the corresponding author upon reasonable request.
